# Morinian Complexity and the Nursing Curriculum

**DOI:** 10.17533/udea.iee.v38n2e11

**Published:** 2020-07-10

**Authors:** Cecilia Rita RÉ

**Affiliations:** 1 Ph.D in Complex Thinking (Philosophy). Adjunct Professor, Universidad Nacional de la Patagonia Austral; Santa Cruz, Argentina. Email: cre@uarg.unpa.edu.ar Universidad Nacional de la Patagonia Austral Universidad Nacional de la Patagonia Austral Argentina cre@uarg.unpa.edu.ar

**Keywords:** education, nursing, nursing care, curriculum., educación en enfermería, atención de enfermería, curriculum., educação em enfermagem, cuidados de enfermagem, currículo.

## Abstract

This scientific essay argues about the relevance of including Edgar Morin's complex thinking in the Nursing curriculum development. The curriculum determines the professional profile of Nursing teachers and future nurses, comprising the cognitive, moral, clinical, affective, research and trade union competencies necessary for professional survival. Complexity allows us to build new creative and critical curricula, with multidisciplinary thinking, connecting fragmented knowledge, defending one's own cultural identity, based on the Philosophy of care, and responding, in parallel, to the challenges of a politically imposed globalization, which is elective. The design of curricula that respond to the demands of the 21st century requires competent curriculum engineering able to triangulate Nursing, Education and Philosophy, given that citizens are been trained and not only specialized workers. The sciences of complexity humanize and complement the Cartesian and positivist scientific approach, influenced by the patriarchal paradigm. The curriculum changes its structure by including complex thinking references.

## Resumen

Este ensayo científico argumenta sobre la pertinencia de incluir el pensamiento complejo de Edgar Morin en la cultura de elaboración de currículos de formación de Enfermería. El currículo determina el perfil profesional de las enseñantes y de las futuras enfermeras comprendiendo competencias cognitivas, morales, clínicas, afectivas, de investigación y sindicales necesarias para la supervivencia profesional. La Complejidad permite construir nuevos currículos creativos y críticos, con pensamiento multidisciplinar, conectando conocimientos fragmentados, con defensa de la identidad cultural propia, basada en la Filosofía del cuidado, y respondiendo, en paralelo, a los desafíos de la globalización políticamente impuesta, la que es electiva. El diseño de currículos que respondan a las demandas del siglo XXI exige ingeniería curricular competente en triangular mínimamente la Enfermería, la Educación y la Filosofía, dado que se forma a personas ciudadanas y no solo a obreros especializados. Las ciencias de la complejidad humanizan y complementan el enfoque científico cartesiano y positivista, influenciado por el paradigma patriarcal. El currículo cambia su estructura por la inclusión de referentes del Pensamiento complejo.

## Resumo

Este ensaio científico argumenta sobre a pertinência de incluir o pensamento complexo de Edgar Morin na cultura de elaboração de currículos de formação de Enfermagem. O currículo determina o perfil profissional dos ensinamentos e das futuras enfermeiras compreendendo competências cognitivas, morais, clínicas, afetivas, de investigação e sindicais necessárias para a sobrevivência profissional. A Complexidade permite construir novos currículos criativos e críticos, com pensamento multidisciplinar, conectando conhecimentos fragmentados, com defesa da identidade cultural própria, baseada na Filosofia do cuidado, e respondendo, em paralelo, aos desafios da globalização politicamente imposta, a qual é eletiva. O desenho de currículos que respondam às demandas do século XXI exige engenharia curricular competente em triangular minimamente a Enfermagem, a Educação e a Filosofia, dado que se forma a pessoas cidadãs e não só a obreiros especializados. As ciências da complexidade humanizam e complementam o enfoque científico cartesiano e positivista, influenciado pelo paradigma patriarcal. O currículo muda sua estrutura pela inclusão de referentes do Pensamento complexo.

## Introduction

“In the same way that light bathes all things and each thing receives and reflects the rays that it is capable of receiving and reflecting, so the inspiring flow of the search for the Path passes through the souls of men and each soul retains what is capable of retaining and reflecting. ”

-Mabel Collins*. Light on the Parh;* 1885-(1)

Since the beginning of the 20th century, the importance of dealing with urgent social issues, including education, health and ecology, was preached in the educational environment, with a futuristic and possibly unintelligible vision for the international political sphere. In the middle of the last century, critical curricular trends rooted in the Frankfurt and Budapest Schools proclaimed a curriculum organized around real-life issues, meaningful to learners and with issues involving democracy. Less than a quarter of the 21st century, bio-psycho-social-spiritual problems are international and, therefore, those of female nurses have become more acute. If the changes have not been implemented in time to avoid their criticality, it is because teachers continue to stumble across old educational schemes when designing their study programs. Edgar Morin opens a promising possibility where the pedagogue has the responsibility to take the risk of walking on other paths and to teach them to the nurses.

### The curriculum pathway

If the heart of the classroom is the lesson thought, programmed, delivered, directed or supervised by the teacher, soul of the classroom, the skeleton that supports the essential knowledge, essential values and fundamental professional and human abilities is the disciplinary curriculum. Scientifically, the educational curriculum is the essential structure supported by a set of criteria such as the study plan, the program, the subjects, the didactic units, the pedagogical models, the methodologies and the processes that contribute to the integral education of future nurses in their knowledge and know-how.

The curricular structure consists of concrete approaches that include four basic elements:(2,3) (i) *Objectives*: they describe the purpose of the program and the subjects answering the question: what is the purpose of teaching? They define the learning objectives of the future nurses. This component needs to be accompanied with the description of the pedagogical approach used. Currently, the objectives of Taylorian origin are called competencies;(4) (ii) *Contents*: they are the information that will be taught during the program and answer the question: what to teach? The contents are selected based on the objectives proposed with the aim of achieving the educational goals stated in the national policies. Philosophies, theories, models, concepts, theoretical, practical and procedural principles such as laws, codes of ethics, techniques, protocols, methods, strategies and health and nursing policies are part of the contents in Nursing; (iii) *Teaching methodology*: it corresponds to the way in which the contents will be approached to achieve the stated objectives. It is linked to the selected pedagogical theory. It answers the question: how to teach? The teaching role and the role of the students are explained. Actions and activities, with the necessary means, resources, place and time are planned. The art and science of the teacher will be revealed in this crucial stage: it represents the critical link between the subject matter and the understanding that the students will make. The appropriate methodology facilitates the learning of the different contents. A technique is neither taught nor learned in the same way as a theoretical notion. With experience, the teacher develops his/her own teaching style and philosophy. The students develop their own learning style; and (iv) *Evaluation*: in this stage the progress of students during a subject are analyzed and measured, and within a study program. Likewise, the quality of the teaching intervention is also analyzed and measured. If the teaching-learning style, the amount of content, the time allocated, the group intellectual rhythm to assimilate the contents have been adequate for the proposed objectives, the adequacy will be reflected in the evaluation results. Frequent evaluations with early communication of their results are recommended. Assessment reinforces the knowledge acquired and motivates learning.

The curriculum acquires instrumental value, conceiving Education as a mere technical process that produces results, that is, nurses strictly aligned to what is programmed, to technical rationality, so that they meet the demands of the local, national and international economy. It is important to highlight that this curriculum is traditionally directed at women, according to Arroyo:(5) “the nursing profession is a female-dominated profession. The act of caring for has been associated in very diverse societies and cultures, and over time, to the female gender. “It is not uncommon to observe that female nurses care and male nurses rule, being the latter those who occupy hierarchical institutional positions(6) sometimes obtained without honesty.

The curriculum is a causal, perpetuating and reproductive means of violence, of oppression of the autonomous critical spirit and of other serious social flaws: it has the tacit objective of suppressing the opposition between genders, between the dominant-submissive relationship, it normalizes people’s capacities affecting work processes in today's society.(7) This curriculum discards the possibility of analysis and criticism by nursing pedagogues.

The curriculum is an educational policy that responds to other cultural policies of colonialism such as those related to gender as well as economic, social, health, environmental and, the almost forgotten humanizing policies. A curriculum must contemplate the indispensable academic, material and physical human resources to implement it. The current trend is to develop cognitive competencies detached from ethical-social values(8) that make societies less rational and relational, more unjust and inhumane with a simulated civilization. The product of this curriculum is creating a society capable of committing the worst atrocities without remorse, demanding from others what it is not capable of giving itself.

### Getting into action

A multifactorial analysis of causes, most of them vertical, rarely horizontal and almost never circular, give rise to the curriculum. Historical, sociological, cognitive, behavioral, and lately, economic reasons determine its form: reality affects the curriculum and its power lies in the fact that it affects reality. During the 1960s Edgar Morin, with his Complex Thinking, produces a titanic intellectual work, philosophically positioned within the Anthropology of Knowledge. His vision implies the reorganization of various theories that emerged in the '40s: those of information, cybernetics, systems, self-organization, among others. His thinking is parallel to the constructivist educational current interested in the students obtaining meaningful learning and that, as Morín said: "... all knowledge today needs to be reflected, recognized, situated, problematized ..."(9) With Morin, the cult to the linear, sequential and rigid is broken, honoring the thinkers of Classical Greece who promoted the need to integrate the dialectics among all the phenomena of reality. Consequently, there is no canonical way to design the curriculum: circularity, helicality, spirality, and unthinkable forms make sense, linking knowledge, process, phenomenon with one another.(10)

The idea of circles, cycles, chains, and unions between processes and actors are close to the real reality. Causality is circular: uninterruptedly effects act on causes and causes on effects.(11) It is the notion assimilated in Morin's Principle of Recursion, where the product becomes a producer. The skeleton of the curriculum is replaced, or, for the most skeptical, supplemented and reinforced by the hologram.

### The nurse, core of the curriculum

When we say *nurse* we refer to the person mastering the science of Nursing, whose aim is to care - the name of the discipline would be a mistake, pointing to what is sick and not to the care to the sick. The name for the science of looking after others will be *Careology* establishing a direct relationship with the preservation and protection of life, which includes ecological health. The logic of the work *La tête bien fait* by Morin refers to a well-planned curriculum focuses on the nurses and the care.

The caregiver is a living system open to their social environment and the curriculum should reflect this aspect: open up to their bio-psycho-social-spiritual human condition, to the knowledge they possess and that they need to incorporate, to social situations which they face with different actors related to their profession. These aspects have an impact on their quality of life, threatening their health and that of their family. The nursing professional has been entrusted with health care in circumstances of poverty, many times being himself/herself in that situation and not only his/her client.

In a Morinian curriculum, the caregiver, the actors and the circumstances interplay. The nurses in the hospital are the bone marrow that supports its functioning and that also functions as a connective tissue because he/she coordinates activities of a varied nature. The real needs and problems that need to be addressed and solved in the field of higher education arise from these aspects. The main aspect in this context is to avoid the perpetuity, recurrence and emergence of problems that affect the nurses (physically, mentally and spiritually) and that have historically been related to gender. World Health Organization and International Council of Nursing report that approximately “90% of the nursing staff are women, but very few managerial positions are occupied by nursing professionals or women. Some data point to the existence of a wage gap between men and women, and to other forms of gender discrimination in the workplace”.(12) The curriculum should approach what ought to be done without forgetting the human nature of the being that you are trying to educate, training him/her so that unjust situations are reversed.

The Morinian curriculum forces to articulate a close communication with different sciences. It is characterized by ideological pluralism. This transdisciplinary basis unites the disunited and humanizes it. Traveling from the limbs to the marrow and from the marrow to the limbs allows us to apprehend the whole and the whole makes it possible to understand the parts.

Nurses are biology, thought, emotion, sensation and action in changing disciplinary settings. Various ideological aspects fall on them, imperfect human being, in their work context: human rights aligned to her gender; public and institutional health policies and those of the healthcare service in which they work; Ergonomics (to preserve part of their health); civil, labor and criminal laws; considerations related to Ethics, Morals and Deontology through regulatory codes of their thinking, attitude and conduct; trade union factors, which may or may not be favorable. Various sciences converge in the nursing curriculum intended for the profession of caring. They should focus on the effector as vehemently as they do towards the clientele. Anatomy is interested in the personal anthropometric and biomechanical aspects, may be affected by professional activity. Physiology represents the metabolic consumption to perform the tasks. The nurse's physiological systems can be functioning perfectly but under a scenario of pregnancy or metabolic disease, her performance and quality of life will be affected. Age is also an impact factor on quality of life in this profession and current working conditions play an unfavorable role leading to early aging and development of diseases.

The mentioned Ergonomics includes the interactions of the future worker with the technical system and with the environment. The Sociology of Work analyzes the fundamental change that workers undergo under the industrial or other model and questions the functioning of the labor groups. The Psychology of Work and Organizations studies the human behavior in contextualized work experiences from an individual, group and organizational perspective. Its objective is to describe, explain and predict these behaviors, but also to solve specific problems that appear in these contexts such as *mobbing* and abuse of power(13,14) with some examples found in teaching.(15,16)

Furthermore, Politics is about empowering the profession so that it achieves greater autonomy(17) but in action, it causes a shrinking crisis in the health sector, impacting on female working conditions.(18) Philosophy in Nursing fulfills several responsibilities within the profession, the axiological ones being paramount, related to human care where the ethic of the act starts on the caregiver. Other sciences are present in the nursing world: Administration, Engineering, Economics, Occupational Medicine, Industrial Hygiene, Safety at work, etc. which, analyzed from a critical perspective and the promotion of rights, would provide personal and professional self-assertion.

### Reorganizing knowledge

The contents are organized around the particular, unique and concrete experiences of caregivers among colleagues, patients, health team, union - an international union of their own would be the most appropriate for this professional group, with 59% of the health professions worldwide,(12) professional organizations, employer sources, the art of caring, at a time, in a space, with a context and with material and physical resources. The sciences that reflect on the preservation of man, planetary life, the social and the human essences enter the field. The sciences are associated with each other and marry divorced cultures, such as holistic therapies, with a little presence in training curricula.

The Morinian position replaces heads full of disjointed contents and void of values with Têtes bien faites, which do science consciously and participate with constructive criticism in building a universally equitable society, which will positively affect the profession. Morin emphasizes the need for intellectual, moral, and affective development. Transdisciplinarity admits chance, uncertainty, holism, progress, chaos, disorganization, order, doubt, error and deviation, indicated by Nicolescu(19) “it moves away from the norm, supposedly indisputable, from effectiveness without brakes and without other values than effectiveness in itself, which is obviously based on the proliferation of academic and non- academic disciplines.”

### Enlightening awareness with science

The dialogue among different types of knowledge propels the cognitive development of nurses. The class integrates theory, research and social interaction. Analysis, criticism and creativity are encouraged in solving and preventing problems. A proportion of content and learning activities are left for students to propose, considering that some of them will emerge spontaneously out of necessity, respecting the position of John Dewey, in which the curricular contents respond to the interests of the learner focused on occupational activities that invite the integrated learning of the disciplines.(20) The daily life of nurses goes from art to science, dealing with professional, personal, collective, local, national and global aspects. James Beane's(21) curricular integration teaching proposal coincides with Morin in its democratic, socialist, progressive and unifying essence.

Nursing is an integral and holistic science per se, totally social, in which holistic and loving care should start from the group itself. The strengthening of personality of the individuality, of the self and of the collective professional image become important. Martin Seligman's Positive Psychology(22) makes a contribution through a curricular design that encourages the development of positive emotions. These make the nurse to be happy, a feeling that has a favorable impact on health, performance and life satisfaction. Social-ethical values become meaningful in the goal of universal happiness.

The Morinian perspective favors cultural identity construction which can be transferred to the concept of caring. Caring has its own nuances according to each geography, that is, it is identified with a nation, with regions, with localities and with personalities. Safeguarding the feelings and doings of Nursing a duty in the face of the advance of intellectual colonization imposed by globalization. Alternative regional and millennial therapies have their space in the Morinian thought, since their care philosophies address "integrity, cross-culturality and even unitary systems",(23) which are also supported by the World Health Organization,(24) which has included these as a strategy to achieve health for all. Diversity is richness and it must be defended. Complexity helps to understand the concept of social inertia, that is, the permanence and repetition of unpleasant and undesirable events for human life, in the local society or at a world scale. Apart from material and intellectual poverty, we have the maintenance of gender violence exerted against nurses. To understand this inertia is to equip nurses with tools to change it inaction: incorporation of feminist theories, Morinian thought, Paulo Freire’s pedagogy of liberation,(25) Karl Marx’s ideas about education,(26) Baruch Spinoza's philosophical argumentation about sad passions like fear, hatred, greed and cruelty that enslave us.(27) Sad affections diminish our potential to act and react. Parnet and Deleuze say that "We live in a rather unpleasant world, in which not only people, but also the established powers have an interest in communicating sad feelings.^"^(28)

### Liberating the curriculum

Traditionally victimized by the influence of positivism-Cartesianism-Taylorism,(29-33) the curriculum displays the following characteristics:

- Abstract with fragmented and disintegrated subjects of the interests, motivations and realities of the actors;
- Programs respond to the labor market and employer interests: it is third party;
- Dictatorial, prescriptive, authoritarian and standardizing;
- Theory and practice form fragmented systems and are, in turn, decontextualized;
- The student seen as a product to be created through homogenizing competences;
- Normalizing teaching of knowledge, behaviors and attitudes; mass production with robotization;
- Neutral, foreign, de-personified contents (colonizing and pressure learning);
- Memorization, assimilation, interpretation of imposed content;
- The teacher is manipulated by policies and sectors interested in producing workers;
- The teacher is the product of that curriculum: absence of critical thinking, conservative obedience; numbness of consciousness;
- Teaching of competences, capacities, contents that serve to enrich others by uncritical doing;
- Suppressed ethical-political discourse: crises, poverty, injustices arise out of nowhere and without responsibility;
- Avoidance of change and revolution, conformist spirit: status quo
- Static scientific content supported by scientific evidence limited to the subject.

Emerging from Complex Thinking, with holistic or integral educational positions, the curriculum becomes an object clearly differentiated from the previous one:

- Meaningful with multi-articulated subjects interested in the needs and individual meanings of teachers and students;
- Programs respond to the interests of the profession and to universal ethical principles;
- Flexible, democratic, liberating: it awakens the consciousness, enhances the human and the unrepeatability of being;
- Articulation between systems, theories and practices;
- Theory and practice at a time, in a place, with a culture, at a specific historical moment, with material, human and financial resources;
- Students conceived in their unique biographical bio-psychosocial-spiritual human conditions, with innate competencies, with rhythms and styles for being, knowing and doing and specific interests that they wish to learn;
- Teaching reinforces individuality in its diversity and reinforces collective strengths;
- Induced contents of the reality of the immediate environment: family, neighbors, group, neighborhood, city, etc.; with socio-historical-cultural relevance, complemented by another optional reality (Genuine and motivated learning);
- Memorization, assimilation, interpretation of content arising and applied from everyday environments, with real actors, linking systems with the idea of cause-effect analysis, incorporating new concepts out of necessity;
- Free and critical teacher interested in the common well-being and happiness for everybody;
- Teaching of competences, skills and contents that reinforce the personal and collective value of the profession, with a focus on being and knowing;
- Ethical-political discourse problematized in the daily care;
- Openness to change and revolutions, emancipatory critical spirit.

Contents energized by new and old knowledge that help to understand, preserve and increase the quality of life, be they scientific, empirical or intuitive, with the incorporation of metaphysics and advances in quantum science and others such as psycho-immuno-neuro-endocrinology, which are a challenge for higher education.

### Liberating cognitive pathways

Nurses’ intelligence is manifested in their cognitive ability to solve novel problems using their previous experience. Nursing educational processes should promote the potential of cognition through the development of natural thinking skills, which works by establishing relationships. Most people think in a suboptimal manner: brain power is wonderfully infinite, but it needs a lot of varied information to ethically solve and create in freedom of action. Restricting the freedom to act kills creativity and intelligence. Educating involves transforming the mind of caregivers and enabling them to survive.(34) Thought modeling facilitates meaningful learning -as Ausubel suggests- by establishing relationships, networks, including new knowledge into existing gears.(35) Thus, uniting, analyzing and synthesizing the cause-effect relationships between organizations, realities, stories, close or distant, through systemic thinking, the caregiver breaks with the dynamics of blindness that oppresses her as a person and as a professional. The dynamics that underlie the problem base is unveiled and from there it is possible to generate changes.

### Time of leisure, time for life

The word *leisure* derives from *school*, therefore, it is "the name by which we call the places where education is carried out, and even higher education, means leisure," Josef Pieper explained.(36) Aristotle, in his Ethics to Nicómaco,(37) says that one of the resources for living is having leisure: it is necessary for contemplative life, to delve into the meaning of life and for knowledge. From this comes “virtue is knowledge” according to Socratic teachings. Aristotle, in *Politics,*(38) mentions that leisure is the cardinal point around which everything revolves. However, the flawed curriculum denigrates this human need that has allowed many discoveries, such as that of Isaac Newton. If the nurse is not producing, doing something, even if that something is unethical and unhealthy for her/himself, she/he is considered lazy. Capitalism turns its face away from great truths of the human condition that do not suit it, even if science, which is applauded, states it otherwise.

Neuroscience research highlight the value of idleness and sleep, for example, humans replenish their energy and maintain their health with 8 hours of sleep, while women need 10 to 12 effective hours. Neuroscientist Andrew Smart(39) argues that doing nothing -really and truly nothing- leads to better brain function, with “innumerable benefits for health: the brain has a network called the default neural network that becomes very active when we are idle and that allows access to the unconscious, creativity and emotions. ” La dolce far niente (the sweet thing about doing nothing) is fruitful: love, wisdom, art, poetry, problem solving and creativity depend on it.

Complex thinking vindicates this precious treasure of humanity. The all-important Theory of Cognitive Load(40) proposed by Sweller, Ayres and Kalyugapara for teaching warns about the damage caused to the body and specifically to the brain through cognitive overload producing chronic stress, which involves other serious health consequences, along with other explanatory theories.(41)

## Conclusion

Capitalist modernity discusses economic progress, associated with the instrumentalization of scientific knowledge, penetrating the heart of education: the curriculum, which disregards the human soul and the fragility of the being of both the person who teaches and the one who educates. Reality shows that world societies experience a marked decline in their quality of life. Nurses suffer the constant negative impact of shrinking public health services and negative labor reforms which ignore their biological and psychological condition, generating situations of institutional violence, accidents, occupational diseases and family disagreement.

Formal education obeys the paradigm of doing for a third party with a philosophical-social framework that forgets to form humans to be and to live, distancing itself from Ethics, deforming the human condition into something for the market. Living to be happy is not even mentioned. Complex thinking rescues knowledge, healthy lifestyles, buried values that can be revived in the curriculum by critical pedagogues through their way of teaching. Humanistic quality knowledge with transfer in the example is part of education. Their absence in the so-called higher education is a shame. These cannot be weak in a curriculum nor in the personalities of teachers.

The future caregiver would be able to respond to her/his needs as a person so as to help with the needs and health problems of others and within healthy work contexts. Empowering future professionals in paradigms that claim the right to a full life must be reflected in caregiver teachers. The deontological obligation forces nurse mentors to review the philosophies, the purposes, the principles and the educational objectives that move away from a high quality of life. Happiness is a unique combination of what Seligman(22) calls “distinctive strengths” such as a sense of humanity, temperance, persistence, and the ability to lead a purposeful life. Nursing pedagogy teaches how to care to preserve life. This is not achieved without taking care of the caregivers and without defending the right to take care of oneself to care.

Applying Complex Thinking brings light to our profession, understood as survival. Good education is the key to success in life. Teachers have a lasting impact on the lives of our students. Rescuing the human, freeing the soul and helping to live with dignity depend on the committed reflection that we make about our teaching practices, which should also be decent.
